# Correlation between dynamic changes in free triiodothyronine levels and 90-day prognosis in patients with HBV-related acute-on-chronic liver failure

**DOI:** 10.1186/s40001-022-00718-8

**Published:** 2022-06-07

**Authors:** Jian Zhang, Yu Chen, Mei Ding, Zhongping Duan

**Affiliations:** Difficult Liver Disease and Artificial Liver Center, Beijing You’an Hospital, Capital Medical University, No. 8, You An Men Outer Street, Fengtai, Beijing, 100069 China

**Keywords:** Hepatitis B, Acute-on-chronic liver failure, Prognosis, Free triiodothyronine

## Abstract

**Background:**

Patients with HBV-related acute-on-chronic liver failure (HBV–ACLF) have a high 90-day mortality rate, so early prognostic evaluation is important.

**Aim:**

We aimed to explore the correlation between dynamic changes in free triiodothyronine (FT3) levels and 90-day prognosis of patients with HBV–ACLF.

**Methods:**

A prospective cohort study investigated 122 inpatients with HBV–ACLF. Patients were divided into three groups based on the dynamic change in FT3 level, as follows: continuous normal FT3; continuous decreased FT3; and FT3 U-shaped change groups, and patients were divided into survival group and non-survival group according to whether they were alive or not. The correlation between the change in FT3 level and 90-day prognosis was explored. Three factors that affected the prognosis most significantly were used to form an FT3 correlation formula to compare the difference in predicting prognosis between the formula score and the conventional score.

**Results:**

There were 98 patients with decreased FT3 level (80.33%), and the lowest FT3 level was at 8.52 ± 6.38 days after admission, which lasted for 16 days. There were no significant differences in FT3 levels at admission and at the lowest point between the survival and non-survival groups. Cox regression analysis showed that the FT3 level, FT3 change type, and hepatic encephalopathy (HE) grading were important factors related to prognosis. The area under the receiver operating characteristics curve for the FT3 correlation formula score was 0.892, which was significantly higher than that of the CTP, MELD, MELD–Na, CLIF–SOFA, CLIF–C OF, and AARC scores (*P* < 0.001).

**Conclusions:**

The FT3 level and its dynamic change type together with the HE grading can facilitate prediction of 90-day prognosis for patients with HBV–ACLF.

## Introduction

Acute-on-chronic liver failure (ACLF) refers to complex clinical syndromes of acute liver function deterioration accompanied by organ failure and high short-term mortality that occurs secondary to chronic liver disease [1]. Although there is a different understanding of ACLF between Eastern and Western countries, it is agreed that ACLF has an extremely high 90-day mortality rate [2, 3]. There is currently no effective treatment that is available except for liver transplantation, and therefore, it is very important to predict the patient prognosis at an early stage.

There are differences in the understanding of the ACLF definition and its etiology between Eastern and Western countries. In Europe and the United States, its primary cause is alcoholic cirrhosis, followed by HCV infection, and the most important cause is bacterial infection and alcohol intake [2]. In the Asia–Pacific regions, HBV infection is the most common etiology, and HBV activation is the main cause of ACLF [3]. The prognostic prediction models that were established in Eastern and Western countries have different evaluation effects [4–6]. The Child–Turcotte–Pugh (CTP) score [7] and the model for end-stage liver disease (MELD) score [8] are used to evaluate patients with liver cirrhosis, but only a limited number of organ functions are considered, so their accuracy has been questioned. The chronic liver failure–sequential organ failure assessment (CLIF–SOFA) [9] and CLIF–consortium organ failure score (CLIF–C OF) [9] mainly focus on the failure of extrahepatic organs based on patients with alcoholic liver disease and hepatitis C, and their accuracy for prognostic assessment in patients with HBV remains to be verified. The APASL ACLF research consortium (AARC) scoring system may be biased and affect the accuracy of prognosis assessment owing to lack of reliable medical evidence.

In addition, the most commonly used prognostic scoring models predict the prognosis of patients based on the degree of organ failure, which leads to a contradiction between the accuracy of model prediction and the convenience of clinical application. CTP score uses five factors (total bilirubin [TBil], HE grading, albumin [ALB], ascites volume, and prothrombin time [PT–INR]) to evaluate patient prognosis. The MELD score uses four factors (TBil, PT, serum creatinine [Cr], and liver cirrhosis category) to assess patient prognosis, while CLIF–SOFA and CLIF–C OF both use six factors (TBil, HE, Cr, PT or PLT, mean arterial blood pressure, and SPO_2_), the AARC uses five factors (TBil, HE, Cr, PT, and LA), and the APACHE-II score uses 14 factors. The accuracy of predicting patient prognosis based on only a few organ functions is often questioned. Increasing the number of organs to be assessed can increase the accuracy of assessment, but collecting clinical data then becomes more difficult, which restricts its clinical application.

Physiologically, humoral regulation is a main regulatory method to maintain homeostasis. When the internal environment changes significantly, the hormone levels change accordingly, and the patient’s condition and prognosis can be predicted based on the hormone levels. In accordance with relevant studies, the low T3 syndrome that is caused by decreased total thyroxine and free triiodothyronine (FT3) can be used to predict the prognosis of critically ill patients with acute myocardial infarction [10], brain tumor [11], and ACLF [11]. HBV–ACLF involves multiple organs, and their functions change significantly. FT3, which is one of the fluids that regulate hormones, may participate in homeostasis regulation in patients with HBV–ACLF. In this study, a prospective cohort observation method was used to explore the correlation between dynamic changes of FT3 levels and 90-day prognosis for patients with HBV–ACLF, to provide a basis to predict 90-day prognosis in patients with HBV–ACLF using FT3.

## Methods

**Fig. 1:**
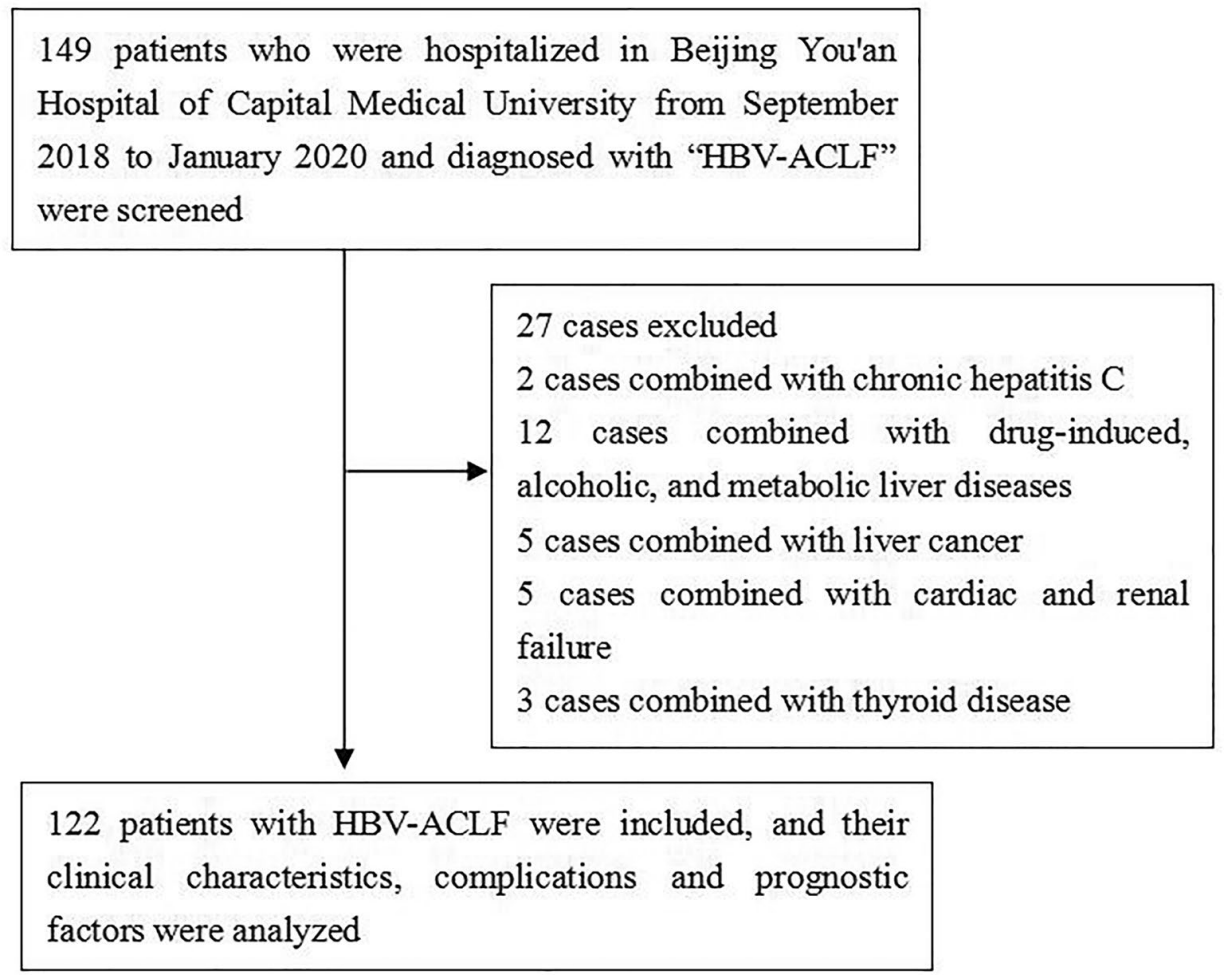
Research flow chart

In this study, 149 patients with HBV–ACLF who were hospitalized in Beijing You’an Hospital of Capital Medical University from September 2018 to January 2020 were selected. HBV–ACLF was defined as the short-term clinical symptoms of acute liver decompensation secondary to chronic HBV infection, with the manifestations of extreme fatigue, obvious gastrointestinal symptoms; rapid deepening of jaundice, serum TBil greater than ten-times the upper limit of normal or a daily increase ≥ 17.1 mmol/L; bleeding tendency with PTA ≤ 40% (or INR ≥ 1.5) with other causes excluded; decompensated ascites; and with or without hepatic encephalopathy [12]. After excluding two patients with concomitant chronic hepatitis C; 12 patients with pure drug-induced, alcoholic, metabolic liver disease; five patients with liver cancer; five patients with cardiac and renal failure; and three patients with thyroid disease, a total of 122 patients with HBV–ACLF were included in this study (Fig. 1).

The patients’ general information, clinical test results, and diagnosis and treatment information were collected, including sex, age, underlying liver disease, and causes of liver failure (such as HBV reactivation, intra-abdominal infection, drinking, upper gastrointestinal bleeding, respiratory infection, and drugs). The patients’ alanine aminotransferase (ALT), aspartate aminotransferase (AST), TBil, PT–INR, HBV–DNA quantification, Cr, ALB, serum sodium (Na), white blood cell (WBC) count, and FT3 were collected. The CTP score [7], MELD score [8], MELD–Na score [13], CLIF–SOFA score [9], CLIF–OF score (Chronic Liver Failure–Organ Failure score) [9], and AARC score [6] were used to evaluate each patient’s condition.

After admission, patients’ FT3 levels were assessed every 4 days. Based on the type of changes in the FT3 levels, the patients were divided into the following three groups: continuous normal FT3 group, FT3 U-shaped change group (FT3 level rose more than 15% after it dropped to the lowest point), and continuous decrease FT3 group (the FT3 level fluctuated or did not rise to 15% after it dropped to the lowest point). Patients were followed-up for 90 days after admission through the outpatient medical record system or telephone, and the 90-day prognostic condition (survival, death, and liver transplantation) after the diagnosis was collected. Patients with liver transplantation were considered to be lost to follow-up.

Statistical processing was performed using SPSS25.0 statistical analysis software. Data that had a normal distribution were presented as the mean ± standard deviation (*X* ± SD). A *t* test was used to compare the means between two groups, and an analysis of variance (ANOVA) was used to compare the means between the three groups, followed by the least significant difference (LSD) test to check for differences between the groups. The multivariate Cox regression analysis and stepwise forward method were used to analyze the factors that affected the patients’ prognosis. The Kaplan–Meier method was used to draw the survival curve, and the log-rank test was used for cumulative survival analysis. The area under the receiver operating characteristics (AUROC) curve was used to compare the prediction differences of various models, and *P* < 0.05 indicated that differences were statistically significant.

## Results

### Patients’ characteristics at admission

One hundred and twenty-two patients were enrolled into this study, including 99 men (81.15%) with an average age of 44.7 ± 9.9 years and 23 women (18.85%) with an average age of 55.2 ± 11.1 years. The patients’ characteristics at admission in the survival and non-survival group patients are shown in Table 1. In terms of factors for acute exacerbation, 45 (36.89%) patients had HBV reactivation, 31 patients (25.41%) had intra-abdominal infection, 16 (13.11%) patients drank alcohol, 12 (9.84%) patients had gastrointestinal bleeding, 10 patients had respiratory infection (8.20%), and 8 (6.56%) patients had exacerbation caused by drugs. There were 69 (56.56%) patients with hepatic encephalopathy, 17 (13.93%) patients with renal failure, 13 (10.66%) patients with lung failure, and 5 (4.10%) patients with circulatory failure.

**Table 1:** HBV–ACLF patient demographic information at admission

	Survival group (77 cases)	Non-survival group (45 cases)	*P* value
Clinical characteristics			
Age (year)	44.65 ± 10.58	48.85 ± 10.13	0.057
Gender(m/f)	64/13	35/10	< 0.001
Chronic hepatitis B/cirrhosis	32/45	14/31	< 0.001
HBV reactivation	30	15	
Intra-abdominal infection	22	9	
Alcohol drinking	10	6	
Gastrointestinal bleeding	6	6	
Respiratory infection	6	4	
Drug	5	3	
Laboratory test			
ALT (U/L)	439.11 ± 239.23	331.48 ± 204.39	0.328
AST (U/L)	348.91 ± 216.14	302.59 ± 194.49	0.642
TBil (µmol/L)	365.14 ± 156.89	432.21 ± 151.34	0.040
PT–INR	2.61 ± 0.86	3.70 ± 0.86	< 0.001
Ln(HBV DNA)	3.82 ± 2.09	3.90 ± 1.52	0.753
Cr (µmol/L)	65.13 ± 28.85	115.03 ± 53.66	0.004
ALB (g/L)	30.44 ± 4.03	29.34 ± 3.54	0.173
Na (mmol/L)	136.30 ± 3.37	133.82 ± 5.73	0.007
WBC (× 10^9^/L)	7.69 ± 3.62	8.63 ± 4.07	0.235
FT3	2.66 ± 0.65	2.43 ± 0.48	0.087
Prognostic score			
CTP	11.54 ± 1.34	12.43 ± 1.44	0.002
MELD	28.10 ± 4.54	32.83 ± 4.68	< 0.001
MELD–Na	28.74 ± 4.32	33.49 ± 4.34	< 0.001
CLIF–SOFA	8.43 ± 1.72	10.46 ± 2.36	< 0.001
CLIF–OF	9.68 ± 1.54	11.14 ± 1.46	< 0.001
AARC	9.33 ± 1.78	10.80 ± 1.23	< 0.001

*ALT* alanine aminotransferase, *AST* aspartate aminotransferase, *TBil* total bilirubin, *INR* international normalized ratio, *Cr* Creatinine, *ALB* albumin, *BUN* blood urea nitrogen, *WBC* white blood count, *FT3* free triiodothyronine, thyroid‐stimulating hormone, *CTP* Child–Turcotte–Pugh score, *MELD* model for end-stage liver disease score, *MELD*–*Na* MELD–sodium score, *CLIF‐SOFA* chronic liver failure–sequential organ failure assessment, *CLIF*–*C OF* CLIF–consortium organ failure score, *AARC* APASL ACLF research consortium

*P* < 0.05 was considered to be statistically significant

All patients received oral antiviral therapy with nucleoside (nucleotide) analogues, including entecavir 0.5 mg/day in 65 (53.28%) patients, entecavir 1.0 mg/day in seven (5.74%) patients, tenofovir disoproxil fumarate 300 mg/day in 25 (20.49%) patients, entecavir 0.5 mg/day combined with tenofovir disoproxil fumarate 300 mg/day in 14 (11.48%) patients, and tenofovir alafenamide fumarate 25 mg/day in 11 (9.02%) patients. In addition to antiviral treatment, conventional treatments included liver protection treatment, plasma and albumin infusion, and nutritional support. Artificial liver plasma exchange was performed in 14 (11.48%) patients. Patients with hepatorenal syndrome or renal failure received albumin, terlipressin, and/or renal replacement therapy; patients with hepatic encephalopathy received treatment with lactulose, ornithine aspartate, and arginine; patients with bacterial infection received antibiotic therapy; and patients with shock received anti-shock treatments, such as blood volume expansion and vasoactive drugs. Patients with respiratory failure were given oxygen therapy, and supportive ventilator treatment was used when necessary. Eight patients (6.56%) received liver transplantation during their hospitalization.

### Correlation between FT3 level and MELD score at admission

The average FT3 level of all patients on admission was 2.59 ± 0.61 pmol/L; 98 (80.33%) patients had a decreased FT3 level, suggesting that a decrease in FT3 level was common in patients with HBV–ACLF. At admission, the mean FT3 level in the survival group was 2.66 ± 0.65 pmol/L and that in the non-survival group was 2.43 ± 0.48 pmol/L. There was no significant difference between the two groups (*P* = 0.087). The correlation between the FT3 level at admission and conventional prognostic score is shown in Table 2. At the time of admission, there was no significant correlation between FT3 level and most conventional prognosis scores in the survival and non-survival groups.

**Table 2:** Correlation between FT3 level at admission and conventional prognostic score

	Survival group	*P* value	Non-survival group	*P *value
CTP	− 0.137	0.261	0.178	0.305
MELD	− 0.199	0.101	0.192	0.269
MELD–Na	− 0.214	0.177	0.124	0.478
CLIF–SOFA	− 0.120	0.324	0.529	0.001
CLIF–OF	− 0.080	0.514	0.496	0.002
AARC	− 0.138	0.259	0.110	0.530

*CTP* Child–Turcotte–Pugh score, *MELD* model for end-stage liver disease score, *MELD*–*Na* MELD–sodium score, *CLIF*–*SOFA* chronic liver failure–sequential organ failure assessment, *CLIF*–*C OF* CLIF–consortium organ failure score, *AARC* APASL ACLF research consortium

*P* < 0.05 was considered statistically significant

### Dynamic changes of patients’ FT3 levels in the survival and non-survival groups

**Fig. 2:**
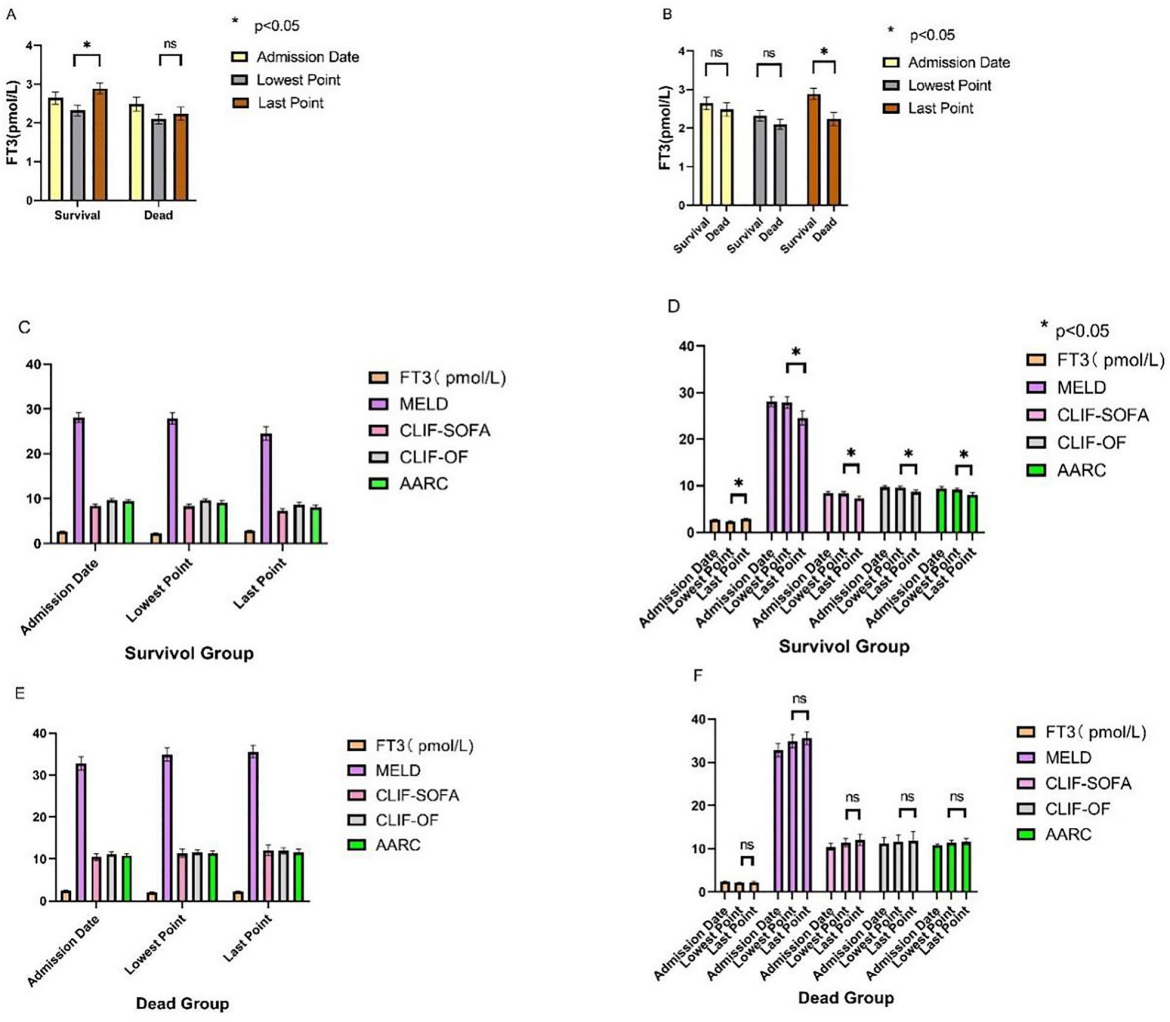
Correlation between FT3 dynamic change and prognostic score. **A**, **B** Characteristics of changes in FT3 at admission, the lowest point, and the end point; **C**, **D** FT3 levels and prognostic score changes in the survival group; **E**, **F** FT3 levels and prognostic score changes in the non-survival group.

Patients’ fasting plasma FT3 levels were measured every 4 days after admission. The lowest FT3 level was at 1–16 days after admission, with an average value of 8.52 ± 6.38 days. The FT3 levels at admission, the lowest point, and the end point within 20 days of admission, and the corresponding prognostic score characteristics in the survival and non-survival groups are shown in Fig. 2A–F. There was no significant difference in the FT3 level between the two groups of patients at the time of admission and at the lowest point. The FT3 level at the end point within 20 days of admission in the survival group was significantly higher than that in the non-survival group (*P* < 0.001). The FT3 level increased in the survival group and the prognostic score was decreased significantly (*P* < 0.01) over time. The FT3 level did not increase significantly in the non-survival group, and the prognostic score did not change significantly (*P* > 0.05) over time.

### Types of dynamic changes in the FT3 levels and patient prognosis

**Fig. 3:**
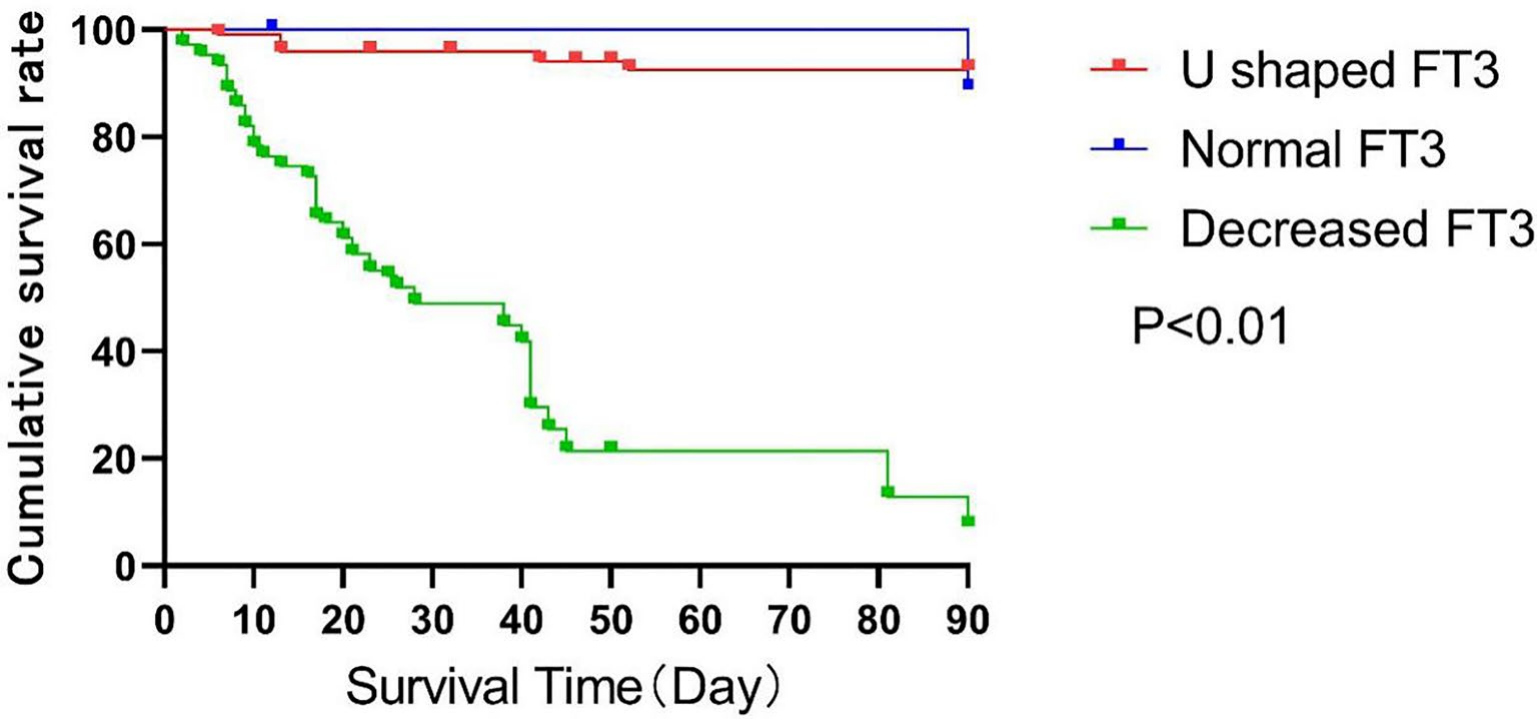
Survival analysis function of patients with different types of FT3 level changes

On the basis of the types of FT3 level changes, patients were divided into the following three groups: continuous normal FT3 group (24 patients, 19.67%), continuous decreased FT3 group (41 patients, 33.61%), and FT3 U-shaped change group (57 patients, 46.72%). There was no other type of FT3 level change in the patients. Seventy-seven (63.11%) patients survived 90 days across the three groups, among whom 24 (100%) patients were in the continuous normal FT3 group, 47 (82.46%) patients were in the FT3 U-shaped change group, and 10 (24.39%) patients were in the continuous decreased FT3 group. The prognosis was significantly different between groups (*P* < 0.001). The patients’ 90-day survival curves are shown in Fig. 3.

### FT3 correlation prognostic formula score

The patients’ 90-day prognosis was used as a dependent variable, and factors including age, sex, underlying liver disease (chronic hepatitis/cirrhosis), FT3 change type, FT3 level, TBil, Cr, eGFR, PT–INR, Na, HE grading, MELD, MELD–Na,

CTP, CLIF–SOFA, CLIF–OF, and AARC score were used as independent variables to establish the COX proportional hazards model. The results are shown in Table 3.

**Table 3:** Multivariate analysis of prognosis with cox regression

	*B* value	Multivariate analysis		
		HR	95% CI	*P* value
Age	0.010	1.026	1.003–1.049	0.025
HE grading	0.320	1.467	1.250–1.721	< 0.001
Type of FT3 level change	− 0.807	0.403	0.322–0.503	< 0.001
FT3 level	− 0.668	0.329	0.217–0.501	0.002
MELD–Na	0.196	1.229	1.172–1.288	< 0.001
CTP	0.004	1.004	0.897–1.125	0.938
CLIF–SOFA	0.015	0.985	0.861–1.127	0.825
CLIF–OF	0.024	1.024	0.854–1.228	0.799
AARC	0.027	1.027	0.919–1.148	0.639

*P* < 0.05 was considered to be statistically significant

The COX regression equation is as follows:

$$Y= 0.010 \, X_1 +0.320\, X_2 - 0.807\, X_3 - 0.668\,X4 +0.196\,X_5,$$

where *X*_1_ is age, *X*_2_ is the HE grading, *X*_3_ is the type of FT3 level change, *X*_4_ is the FT3 level, *X*_5_ is the MELD–Na.

The three factors that had the greatest influence on the prognosis (HE grading, type of FT3 change level, FT3 level) were used to form the following FT3 correlation prognostic formula: *Y* = 0.283*X*_2_ − 0.909*X*_3_ + 0.334*X*_4_. Based on the formula, the FT3 correlation prognostic score was defined (if the FT3 level was continuously normal, the value was 1; if the FT3 level was in a U-shaped change, the value was 2; and if the FT3 level was continuously decreasing, the value was 3).

### FT3 correlation scores and patient prognosis

**Fig. 4:**
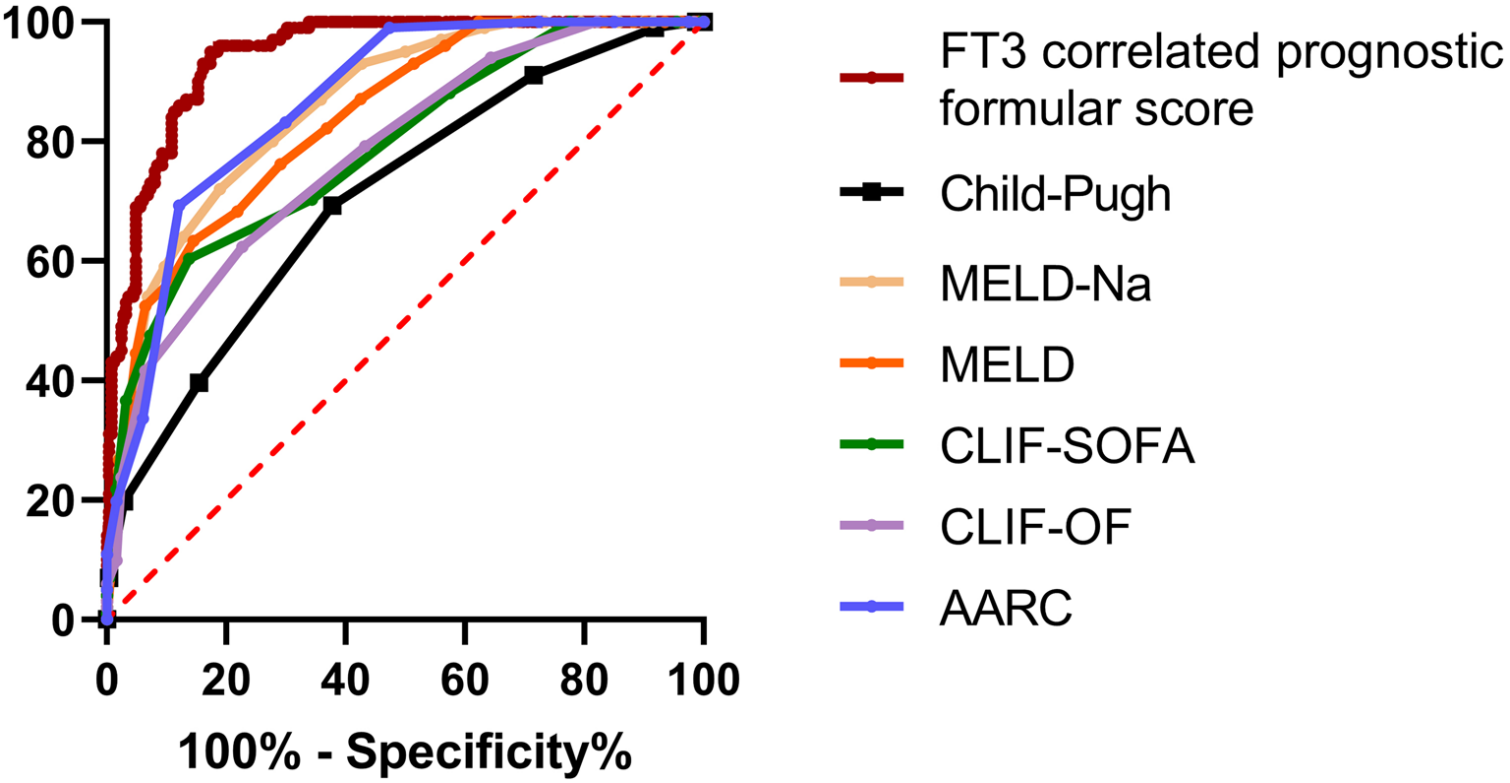
Comparison of the area under the ROC curve between the FT3 correlation formula score and the conventional prognostic prediction model in predicting the accuracy of 90-day mortality rate

The FT3 correlation prognostic formula score was 1.92 ± 0.86 in the survival group, which was significantly lower than that in the non-survival group (4.91 ± 1.27, *P* < 0.001). The ROC curve was used to compare the FT3 correlation prognostic formula score and the AUCs of CTP, MELD, MELD–Na, CLIF–SOFA, CLIF–C OF, and AARC scores, as shown in Fig. 4 and Table 4. The AUROC curve for the FT3 correlation prognostic formula score was 0.944 (95%CI 0.923–0.966), which was significantly higher than that of CTP, MELD, MELD–Na, CLIF–SOFA, CLIF–C OF, and AARC scores (*P* < 0.001).

**Table 4:** Comparison of different prognostic models in predicting 90-day prognosis of patients with HBV–ACLF

	AUROC	95% CI	*P* value
FT3 correlation formula score	0.944	0.923–0.966	≤ 0.001
CTP score	0.707	0.652–0.763	≤ 0.001
MELD score	0.823	0.779–0.867	≤ 0.001
MELD–Na score	0.847	0.806–0.888	≤ 0.001
CLIF–SOFA score	0.787	0.736–0.838	≤ 0.001
CLIF–C OF score	0.778	0.727–0.828	≤ 0.001
AARC score	0.846	0.807–0.885	≤ 0.001

*P* < 0.05 was considered to be statistically significant

### Discrimination and calibration of FT3 related formula scores

**Fig. 5:**
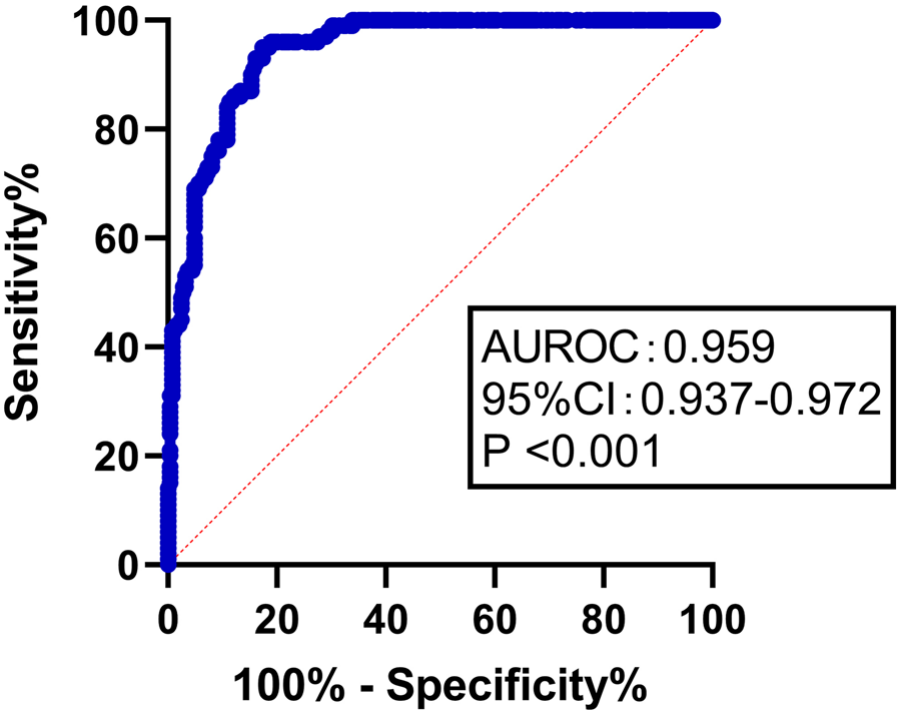
ROC curve of prediction probability in FT3 correlation formula score

**Fig. 6:**
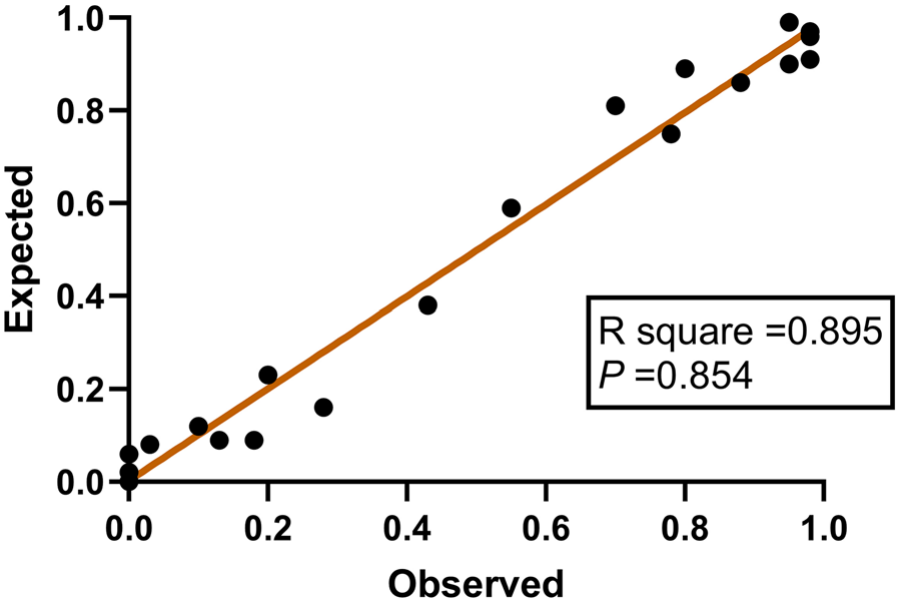
Scatter plot of observed and expected value

ROC curve of prediction probability value was drawn to verify the differentiation of FT3 related formula score, AUC was 0.959 (0.937–0.972), indicating that the prediction model has good discrimination ability. At the same time, the goodness of fit test was used to evaluate the calibration ability of the prediction model. The results showed that R2 = 0.979, *P* = 0.871, indicating that there was no difference between the predicted value and the actual observation value of the model, and the prediction model had good calibration ability. (Figs. 5 and 6).

## Discussion

The synthesis, metabolism, and secretion of thyroid hormones and the synthesis of thyroxine-binding globulin are closely related to the liver [14]. The liver plays an important role in thyroid hormone metabolism, and liver disease can affect thyroid hormone metabolism. ACLF is a disease that occurs secondary to chronic liver disease with acute massive necrosis of liver cells, leading to severe damage to the liver functions, such as detoxification, metabolism, synthesis, and biotransformation [1]. This damage to liver cells may also affect the thyroxine level. Earlier studies have indicated liver diseases induce several abnormalities in thyroid function tests, which is probably a result of low serum T3, normal to low T4 and normal TSH [15, 16]. Wu et al. indicated serum T3, T4, FT3, FT3/FT4, and TSH concentration was significantly decreased in patients with HBV–ACLF. ACLF causes massive liver cell death, it may be the cause of the altered metabolism of T4 and its metabolites. More than 80% of serum T3 is produced by peripheral deiodination. Due to the impaired conversion of T4 to T3 in the liver, parenchymal liver damage in ACLF patients may decrease T3 production [17].

In this study, we found that the FT3 level decreased in 80.33% of patients with HBV–ACLF, which indicates that the decrease in the FT3 level was very common. This laid a foundation for predicting the prognosis of patients with HBV–ACLF using the FT3 level. Based on relevant studies, patients with liver failure of various etiologies had significantly lower FT3 levels than those with chronic hepatitis [11], which were consistent with our results in this study. The main reason was that patients with ACLF produced low levels of thyroid 1-deiodinase because of massive necrosis of liver cells. Inflammatory factors (such as interleukin-6 and tumor necrosis factor-α) decreased the deiodinase activity, and partially inhibited the conversion [14] of TT4 to TT3, and the production of TT3 decreased, which resulted in a decrease in FT3 level.

In this study, there was no significant difference in FT3 levels at admission between the survival and non-survival groups, and there was no significant correlation between FT3 levels at admission and most prognostic scores, suggesting that it is difficult to predict 90-day prognosis for patients with HBV–ACLF using only FT3 levels at admission. The fasting plasma FT3 levels were measured every 4 days after admission. There was no significant difference in FT3 level at the lowest point between the survival and non-survival groups, but the FT3 level at the end point within 20 days of admission in the survival group was significantly higher than that in the non-survival group (*P* < 0.01). In addition, with the increase in the FT3 level, the prognosis score in the survival group was improved significantly (*P* < 0.01), while the FT3 level at the end point did not increase and the prognosis score did not change significantly (*P* > 0.05) in the non-survival group. This suggested that the dynamic changes in the FT3 levels may influence the prediction of 90-day prognosis in patients with HBV–ACLF. On the basis of the type of FT3 level change, patients were divided into a continuous normal FT3 group, a continuous decreased FT3 group, and a FT3 U-shaped change group. The prognosis for patients in the three groups was significantly different, which further suggested that the type of FT3 change may become an important factor in predicting the prognosis of patients with HBV–ACLF. Based on relevant studies, the decrease in TT3 and FT3 at admission could be used to predict the prognosis of critically ill patients with acute myocardial infarction [10], brain tumor [11], and ACLF [11], which was different from this study. The possible cause is that different organ diseases have different effects on the synthesis, metabolism, transport, and secretion of thyroid hormone. The relevant mechanism needs to be further clarified.

Our research based on the Cox proportional hazards model, and introduced three factors (HE grading, type of FT3 level change, and FT3 level) that had the most significant influence on patients’ 90-day prognosis to form an FT3 correlation prognostic formula. The formula score showed that the AUROC curve predicted the 90-day prognosis for patients, which was 0.892, and it was significantly higher than that of the CTP, MELD, MELD–Na, CLIF–SOFA, CLIF–C OF, and AARC scores. The results showed that the FT3 level and type of FT3 dynamic change, combined with HE grading, could be used to predict 90-day prognosis of patients with HBV–ACLF, which may provide new ideas for predicting 90-day prognosis of patients with HBV–ACLF.

Previous studies [18, 19] have shown that critically ill patients showed a reduced metabolic rate and energy loss via decrease in the thyroid hormone level, which helps the body to respond to stresses. Low thyroxine levels have a protective effect on the patients. However, in this study, there were 24 (100%) patients who survived in the continuous normal FT3 group, 47 (82.46%) patients who survived in the FT3 U-shaped change group, and 10 (24.39%) patients who survived in the continuous decreased FT3 group, suggesting that the lower the FT3 level, the higher is the mortality of patients with HBV–ACLF, which does not support the conclusions of previous studies. Further studies are required to elucidate the relevant mechanism.

We metabolic regulatory factors combined with functions of a few organs were used to evaluate the prognosis in patients with HBV–ACLF. These factors reduced the amount of collected clinical data and achieved a better prognostic evaluation effect, which provided new ideas for predicting prognosis in patients with HBV–ACLF. In addition, our research also found significant differences in clinical parameters (TBil, INR) between the survival group and the non-survival group. In addition, these indicators affect the MELD–Na score. Therefore, we believe that changes of FT3 levels may also affect clinical parameters. Patira et al. showed that TBiL, PT, INR, TSH level increases and FT3 level reduces as the severity of liver increases [20].

Klein et al. [21] reported that “during acute infection inflammatory mediators released in the blood stimulate the conversion of T4 to T3 in the tanycytes of the third venctricle.” Therefore, a low level of T3 may be useful in these circumstances by decreasing the metabolic activity of the body. This low metabolic activity allows the conservation of energy when the body should be at rest and avoid dispersion of the energy in a time when all capabilities should be kept for the recovery process from the infection. The immune system will provide the signal from TSH leading to a recovery of metabolic activity. However, in this study, the results did not presented this phenomenon which should be further research in the future.

There are some limitations in this study. First, the accuracy of the FT3 correlation formula score in predicting the prognosis of patients with HBV–ACLF requires validation from other centers in larger group of patients with ACLF (Large sample size with all type of ACLF). Second, the scoring model that was established in this study is based on the Chinese ACLF diagnostic criteria, and the applicability of these results to patients with ACLF in other countries and regions needs to be verified through further studies.

In conclusion, the FT3 levels and type of FT3 dynamic changes combined with HE grading could be used to predict the 90-day prognosis of patients with HBV–ACLF.

## Data Availability

The data sets used or analyzed during the current study are available from the corresponding author on reasonable request.
